# Progress in Surgery

**Published:** 1898-05-14

**Authors:** 


### PROGRESS IN SURGERY.

### NEW GROWTHS.

(*Continued from page* 96.)

**Carcinoma of the Breast** (*continued*).—Dr. J. B. Roberts,[16] before the Academy of Surgery of Philadelphia, has recently advocated amputation of the upper extremity in recurrence of malignant disease after excision of the breast. He had been impressed with the futility of endeavouring to dissect away all the diseased tissue when recurrence took place at the top of the armpit around the axillary artery and vein. It is not sufficient often in these cases to remove the affected portion of vein, for the artery is often involved to an equal degree, and removal of both artery and vein would be very risky, and in the end not so likely to rid the patient of all the growth. In the discussion which followed, the general feeling was that the amputation should only be performed as a last resort.

John F. Binnie[15] in a brief article discusses the reasons why recurrent carcinomata are often locally worse than the primary growth in the same region. He alludes to the mode of spread of carcinoma of the mamma. Normally the lymph stream from the mamma passes chiefly to the axilla, but portions of the stream pass *(a)* with the perforating branches of the internal mammary artery into the mediastinum; *(b)* across the middle line of the body to the opposite side—(Gerota has shown by injection experiments that there is a free anastomosis of lymphatics across the middle line); *(c)* downwards towards the inguinal glands. A leash of lymphatics taking this course were demonstrated by Gerota. They followed the line of the superior and deep epigastric arteries.

When the primary growth of cancer is removed, large numbers of lymphatic channels are obliterated. Probably, therefore, the region is subsequently drained efficiently by the enlargement of the lymphatic channels just described, just as after ligation of a large blood vessel subsidiary vessels enlarge. If cancer recurs these numerous enlarged lymphatics, passing in various directions, serve to disseminate the growth widely, and hence the rapid spread of the cancer over a large area of the chest wall. Some primary cancers show a similar disposition to spread which Binner attributes to the early blocking of the axillary glands and lymphatics by early infiltration. The writer also thinks that a similar enlargement of subsidiary lymphatic routes from the stomach explains why occasionally gastric carcinoma leads to secondary carcinoma at the umbilicus, the spread being along channels running with the round ligament of the liver.

Dr. Dowd,[18] of New York, has made a contribution to the literature of mammary cancer and published records of twenty-nine cases. He points out how statistics show that the efficacy of operations for this disease has improved. Thus, in 1878, von Winwarter found that of 143 cases operated upon only 8 3 per cent. had survived the three-year limit without recurrence. Seventeen years later, Curtis summarised the results of 1,213 cases which had been reported between 1888 and 1893 in Europe, and 22 per cent. remained free after three years' interval. Dowd then gives a table compiled from the published cases of six surgeons of a date subsequent to those in Curtis's paper. Of these last cases, 39·6 per cent. were free after three years. The mortality of the operation has also diminished most remarkably. Weir has reported 125 and Halstead and May each 76 consecutive operations without a single death. Only six of Dowd's cases have yet passed the three-years' limit. Of these three remain free from recurrence. The other three have had recurrence. Dowd then refutes some of the objections which have been raised against the modern extensive operation. He shows that a very useful arm is left even after removal of the pectorals, that there is no permanent œdema of the arm, and that these operations are as safe as any operation in which the axilla is opened up.

Dr. William B. Coley[17] has put on record a case in which a woman 53 years of age had a mammary scirrhus, and also a round-celled sarcoma in the submaxillary region. The sarcoma appeared about 18 months after removal of the scirrhus, and was itself subsequently removed twice, and a recurrent carcinoma was also removed in the mammary region. The writer quotes Roger Williams as having collected eleven examples of the co-existence of mammary cancer with sarcoma elsewhere. In eight of these the sarcoma was in the other breast; in three in other regions.

**Branchial Carcinoma.**—Dr. C. A. Powers, of Denver,[18] records a case of this disease. The patient, a lady of 48, presented herself with a hard swelling on the right side of the neck. It had been growing a year, was the size of a pigeon's egg, irregular, and was deeply seated beneath the angle of the jaw, stretching backward under the sterno-mastoid. The skin was free. No primary growth in ear, nose, mouth, pharynx, or œsophagus was detected. The tumour was removed, and found to be a carcinoma. A year later a recurrent nodule, also carcinomatous, was removed.

At the present time, six years since the first operation, she is free from relapse. The original growth was probably a branchiogenous carcinoma. Dr. Powers points out that Volkmann in 1882 first drew attention to these tumours, and described three cases. Von Volkmann says that these tumours undoubtedly originated from epithelium left behind in the closure of the branchial clefts, and calls them "branchiogenic carcinomata." The writer then gives a brief note of each of nineteen cases, which he has gathered from literature. Eighteen of these were in men, and all the patients with one exception were over 40.

**Epithelioma of the Face with Unusual Infection of Lymph Glands.**—An interesting case of the above nature is recorded by Dr. D. W. Montgomery,[19] of San Francisco. The patient, a woman of 56, presented herself with an ulcer on the right side of the nose, far up near the eye. The ulcer was removed, and microscopically proved to be an epithelioma. About two years later the patient returned with partial facial paralysis, due to a tumour lying over the right parotid. There were also three lumps under the chin, obviously metastases in the submental lymph glands. The patient died three and a half years after the original operation. There was no recurrence at the site of operation. It is not clear that anything could have been done to avert the unfortunate result. It is very rarely that small skin cancers affect lymph glands. Also the glands one would have expected to be involved escaped in this case, viz., the glands under the body and angle of the jaw. When a skin cancer encroaches on mucous membrane, it then frequently shows the same tendency to metastasis as a cancer originating in the mucous membrane.

[16] Annals of Surg., Jan. [17] Annals of Surg., Feb. [18] Annals of Surg., Mar. [19] Annals of Surg., Jan., 1898.

### GENERAL SURGERY.

**Antiseptics.**—In connection with the recent advocacy of the use of picric acid in the treatment of burns, Walther[1] calls attention to two cases of poisoning with this drug. They occurred in children, eleven and four years old. On the first application of compresses wet with picric acid solution there was great pain, lasting half an hour; and on the second dressing, five days later, the pain recurred, and a 10 per cent. picric acid ointment was substituted. The ointment used in each case contained 300 grains of the acid. There was severe sweating, and at the end of 24 hours vomiting set in, and lasted an entire day, with colic, diarrhœa, an intense yellow colouration of the skin, somnolence, prostration, and scanty, dark-coloured urine. On a second application of the ointment the vomiting reappeared, to subside only on the removal of the dressing. In studying the mechanical condition attending the absorption of iodoform, Dr. W. Hubener[2] finds that finely-powdered iodoform is more quickly absorbed and diffused by the lymph channels than the coarser crystals, and that when used in the peritoneal cavity it has a distinct tendency to produce an inflammatory process, resulting in an excessive formation of adhesions. Prior to its ultimate breaking up into its chemical components iodoform undergoes a change into complicated iodine compounds, whose exact nature are as yet unknown. Terson[3] reports an example of partial optic atrophy from the use of iodoform in a case of burn of the thighs and abdomen. After three weeks' use of this drug, a progressive amblyopia appeared, accompanied by atrophy of the temporal half of both discs, which persisted for several years. Iodoform has resulted in double optic atrophy after its application to wounds, and when given internally. The *Lancet*[4] draws attention to the fact that in these days of antisepsis, successful operating, and anti-toxins, the profession must not overlook the pathology and treatment of extensive burns. The lesions found by the valuable researches of Dr. Bardeen, of Baltimore, in the lymphatic tissues, were remarkable for their wide distribution throughout the body, their severity in spite of the few hours elapsing between the burn and the death, and their nature. Lymph sinuses and vessels were found greatly damaged, but the lymphatic glands suffered most severely. Dr. Bardeen[5] also points out that the lymphatic changes must not only imply mechanical mischief, but also represent general infection. He insists that the changes seen in all the internal organs are very similar to those observed after fatal cases of certain fevers, the main effects of which are ascribed to toxic substances circulating in the blood. Thus these lesions of the lymphatic tissue furnish most important additional evidence of a toxæmia after superficial burns. Dr. E. O'N. Kane[6] recommends vinegar as equal in antiseptic power to a 1 in 2,000 bichloride solution, and far superior in its freedom from toxic properties. It is also less irritating to the tissues than mercury. It stimulates the healing process in open wounds instead of retarding it, as bichloride sometimes does. When not too far diluted, if used during an operation, it reduces the free capillary flow by its astringent properties. This author also suggests that it may be used for disinfecting purposes, for floors of operation-rooms, surgical wards, &c.

[1] Gazette hebdomadaire de Méd. et de Chirur., January 27, and N.Y. Med. Jour., March 5, p. 327. [2] Beiträge zur Klin. Chir. Bd., xviii. Heft 1, and Annals of Surgery, Oct., 1897, p. 512. [3] Epit. Brit. Med. Jour., Feb. 19, 1898, and Annales d'Oculist, Nov. 1897. [4] Lancet, Feb. 5, p. 386. [5] Journal of Experimental Medicine, N.Y., Sept., 1897. [6] Railway Surgeon, Vol. IV., No. 3, 1897.

